# *In situ* reconstruction with autologous graft in the treatment of secondary aortoenteric fistulas: A retrospective case series

**DOI:** 10.1016/j.amsu.2019.11.020

**Published:** 2019-12-06

**Authors:** Claudio F. Feo, Giorgio C. Ginesu, Antonio Pinna, Francesca Galotti, Panagiotis Paliogiannis, Alessando Fancellu, Alberto Porcu

**Affiliations:** Department of Medical, Surgical and Experimental Sciences, University of Sassari, 07100, Sassari, Italy

**Keywords:** Aortoenteric fistula, Aortic aneurism, Aortic repair, Autologous graft

## Abstract

Infections caused by secondary aortoenteric fistulas (SAEF) may be extremely complex and threaten patient's life. We report our surgical approach to SAEF consisting in removal of the infected graft and *in situ* reconstruction using an autologous venous graft.

Seven consecutive patients with SAEF treated with graft removal and *in situ* reconstruction using an autologous venous graft from 2008 to 2017 were reviewed. Six of seven patients (86%) survived 30-day. In one case a graft thrombosis and acute lower limb ischemia occurred requiring re-operations. All patients received injective antibiotic therapy for 20 days, followed by oral therapy for 3 months. There were no major complications at long-term follow-up.

Our results suggest that superficial femoral vein reconstruction of the abdominal aorta for SAEF is effective with an acceptable in-hospital mortality and low rate of major complications. We stress the importance of the deep femoral veins to create the graft because the large saphenous vein is often affected by significant intimal hyperplasia that can cause steno-occlusive complications.

## Highlights

• In situ reconstruction for secondary aortoenteric fistulas is effective.
• We stress the importance of the deep femoral veins to create the graft.
• One of the largest series of secondary aortoenteric fistulas treated with venous graft.
• Morbidity and mortality are acceptable at long-term follow-up.

## 1 Introduction

Primary aortoenteric fistulas (PAEF) are very rare entities that are usually caused by erosion of an aortic aneurysm into the duodenum [1,2]. Secondary aortoenteric fistulas (SAEF) are complications of aortic grafting surgery and they are relatively more frequent. Infection of prosthetic vascular grafts is a difficult challenge for vascular surgeons. In particular, infections caused by SAEF may be extremely complex and threaten patient's life. Aortic graft excision and staged or simultaneous extra-anatomic bypass is the historical gold standard of SAEF repair, however this procedure is associated with a very high morbidity. Several different *in situ* reconstruction techniques have been developed over the years, but presently no definite conclusion can be drawn.

As the treatment of choice for SAEF is still under debate, we describe our surgical approach to SAEF consisting in removal of the infected graft and *in situ* reconstruction using a novel autologous venous graft.

## 2 Materials and methods

### 2.1 Patients

The medical records of seven consecutive patients with SAEF treated at our academic university hospital, from 2008 through 2017, with removal of the infected graft and *in situ* reconstruction using an autologous venous graft were reviewed, and the demographic and clinical data were analyzed. All procedures were performed by the same experienced general and vascular surgeon.

An institutional board (Comitato Etico, AOU Sassari) approved this retrospective case series and informed consent was obtained from all patients prior to each procedure. This study is compliant with the PROCESS 2018 guidelines [3] and has been registered in the Research Registry (Unique Identification Number 5210).

### 2.2 Surgical technique

A midline xifo-pubic laparotomy and adhesiolysis were performed in order to access the retroperitoneal space. The bowel segment involved in the fistula was identified and a blunt dissection was carried out to find a correct cleavage plane between the intestinal wall and the prosthesis.

**Fig. 1:**
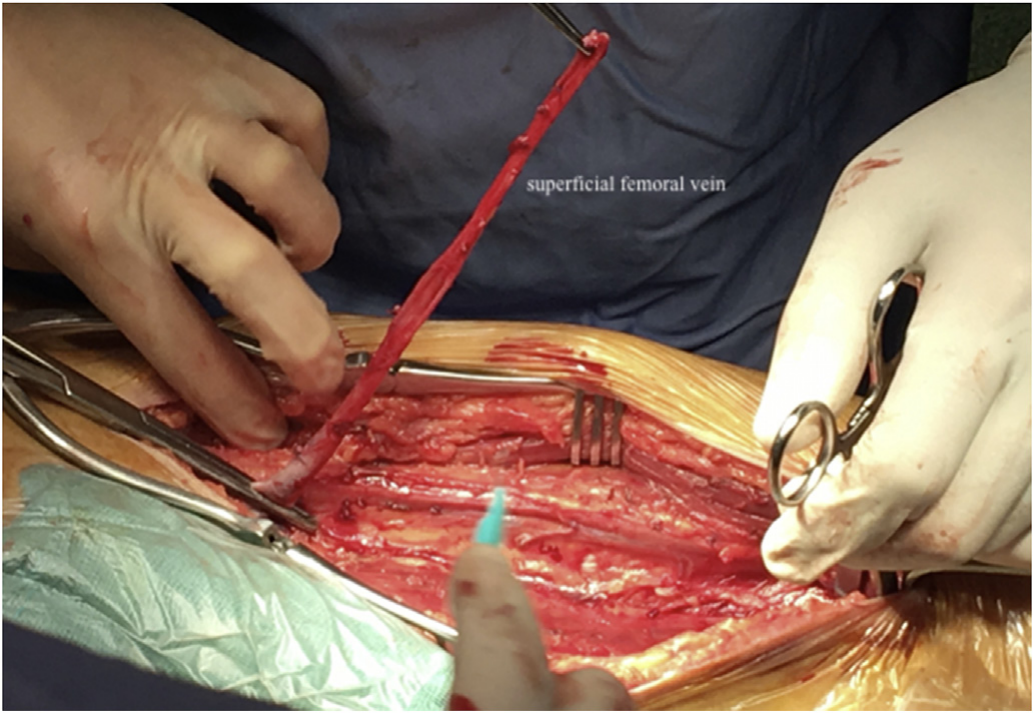
Harvesting of the superficial femoral vein.

**Fig. 2:**
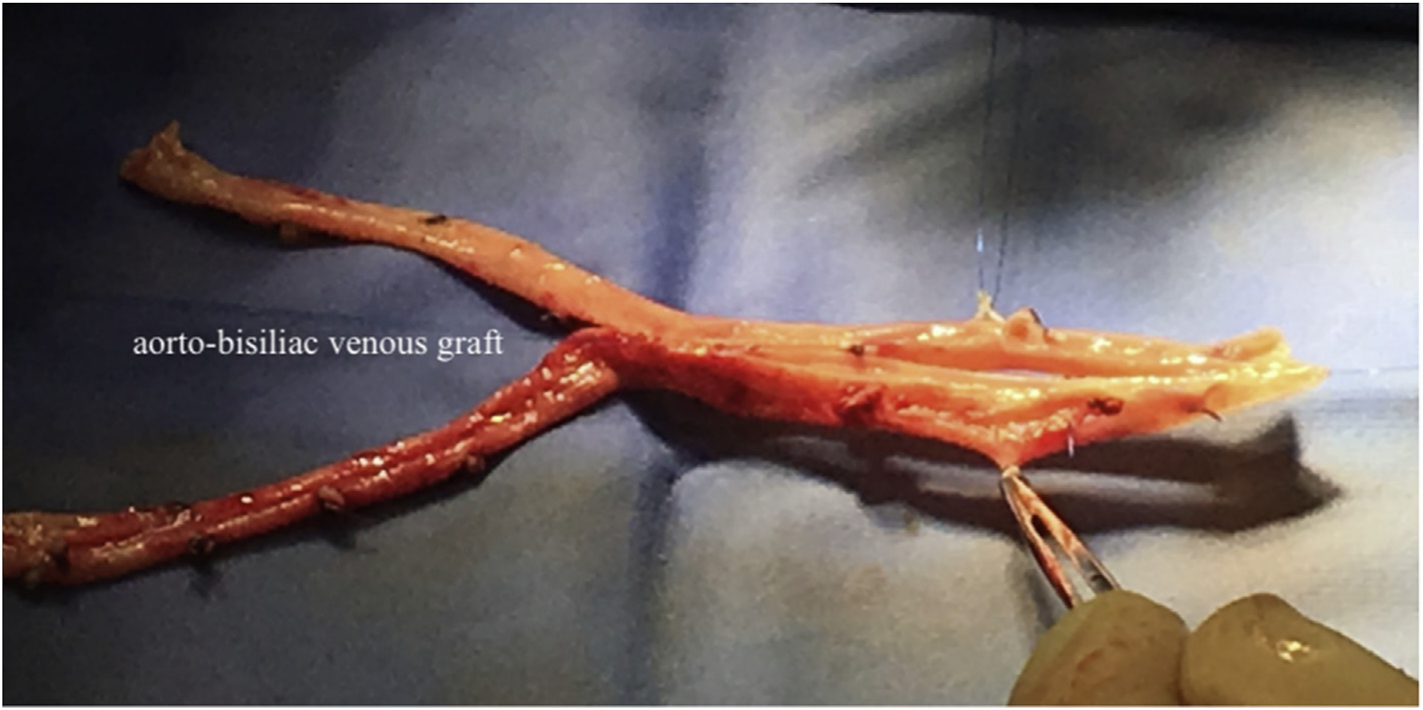
Construction of an aorto-bisiliac venous graft.

The type, shape and dimensions of the required autologous venous graft were assessed before proceeding with the removal of the infected prosthesis. A 15- to 20-cm tract of the superficial femoral vein was isolated, reaching the confluence with the common femoral vein and preserving the deep femoral branches; the superficial femoral vein was sectioned, only after ligation and section of its confluents (Fig. 1). The venous segment was reversed and dilated, and two longitudinal venous segments were used to create an aorto-bisiliac/bifemoral graft, after venotomy for about 2/3 of the length of the graft (Fig. 2). When a straight aortic graft was required, the venotomy was performed for the entire length of the vessels, which were then sutured to get a straight venous graft. The graft was kept in sterile saline after hydropneumatic testing of the sutures.

**Fig. 3:**
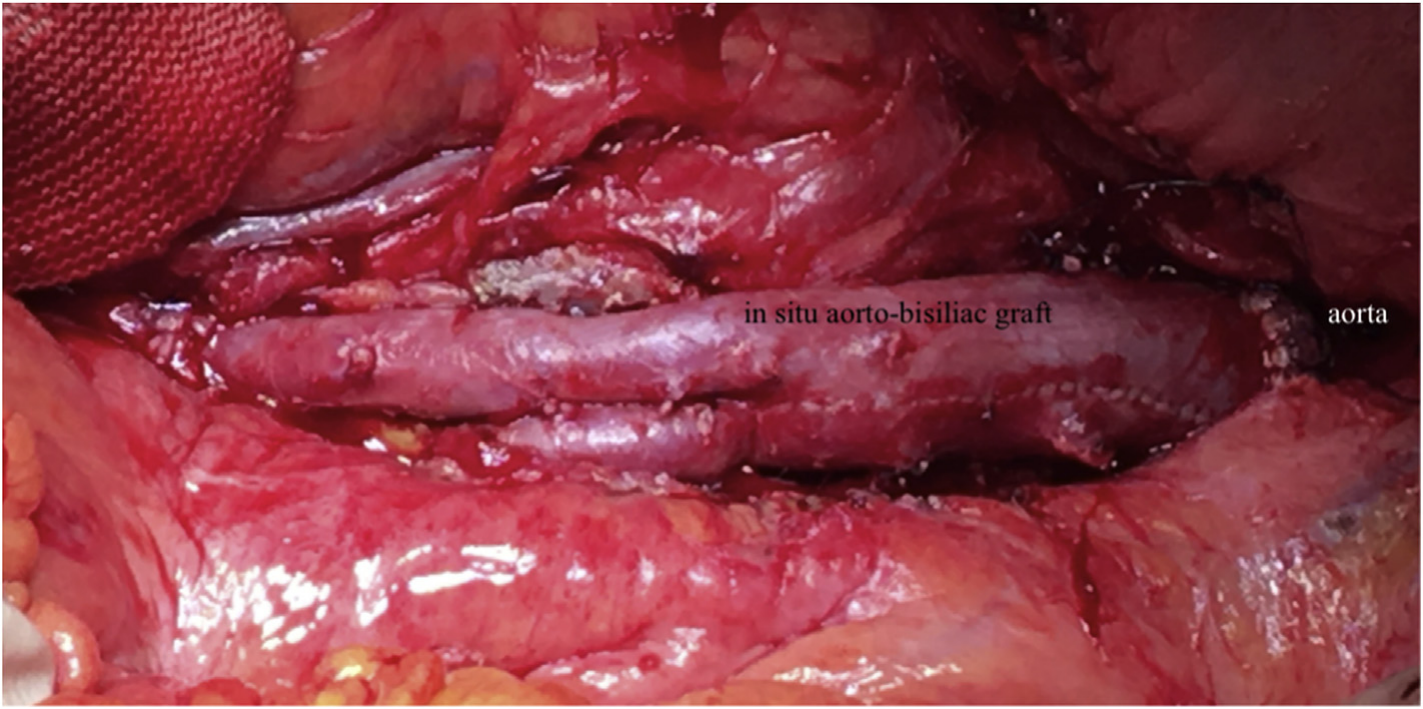
*In situ* autologous venous graft.

The next step was the isolation of the bowel from the infected prosthesis. After accurate debridement, the intestine was closed with a running monofilament suture. Aorta and iliac/femoral clamping were performed with previous heparinization, and the infected prosthesis was excised. The next step was removal of any residual infected or necrotic tissue, including the aortic margins and the retroperitoneal inflammatory tissue. The excised tissue was sent for microbiological examination in all cases. Subsequently, a proximal end-to-end anastomosis was performed, and after anastomotic leakage testing the remaining sutures were completed (Fig. 3). Once anastomotic patency, bowel viability, and lower limbs perfusion were assessed, the proximal suture was protected with an omental flap. A retroperitoneal drainage tube was placed before closing the laparotomy.

## 3 Results

All the seven patients identified were men with a mean age of 69 (range 62–73) years, who had undergone surgery for a sub-renal aortic aneurysm. The previous surgery was aorto-aortic replacement in four (57%), aorto-bifemoral in two (29%), and aorto-bisiliac in one case (14%). Repair of the aortic aneurysm was performed with a dacron prosthesis in all cases. Six patients were affected by hypertension, one had pancreatic cancer, one other had chronic obstructive pulmonary disease (COPD) and previous surgery for bowel obstruction; finally, one patient suffered from rheumatoid arthritis. There was hematemesis in 6 cases (86%), melena in 5 (71%), and abdominal pain in 3 (43%). The mean preoperative hemoglobin value was 11.8 (range 7.3–15.0) g/dL.

A computed tomography angiography (angio-CT) was performed in all cases, as well as a doppler ultrasound (US) to evaluate the superficial femoral veins. A gastroduodenoscopy (EGDS) was carried out in 6 patients (86%). One patient underwent traditional angiographic examination.

The mean time lapse between the prosthesis placement and repair with autologous graft was 78 (range 23–192) months. All patients were sent to the intensive care unit (ICU) postoperatively for a mean stay of 10 (range 3–25) days. Six patients (86%) received blood and plasma transfusions. Postoperative heparin prophylaxis was given in all cases until full mobilization or hospital discharge. The 30-day survival was 86% (6/7). In one case (the patient with the largest number of comorbidities) a graft thrombosis and acute lower limb ischemia occurred requiring re-operations, but the patient died in the 25th postoperative day. All patients received injective antibiotic therapy for 20 days based on the results of antimicrobial susceptibility testing, followed by prophylactic oral therapy for three months.

All patients were observed 6 months after the operation and then at 1-year intervals, and they were evaluated with vascular physical examination and doppler US. Further examinations (blood tests, abdominal US, angio-CT) were performed as needed. The mean postoperative follow-up time was 63 (range 9–125) months. There were no major complications; a mild bilateral lower limbs edema occurred in one case and was treated with elastic stockings.

## 4 Discussion

Brock [4] first described the presence of a fistula between an aortic graft and the duodenum in 1953, where as the first SAEF surgical repair was carried out by Mackenzie [5] in 1958. SAEF are very rare, accounting for less than 1% of the complications related to aortic surgery with prosthetic material.

The goals of SAEF surgical treatment are control of bleeding (if present), removal of the prosthesis and any peri-prosthetic tissue, and restoration of bowel and vascular continuity. The most used vascular reconstruction method is the extra-anatomic bypass [6], which accounts for approximately 10% limbs’ amputation, 15% re-infections, 18% thrombosis, and 15% aortic suture failure [7,8] with perioperative mortality up to 23–44% [9,10]. For this reason, the *in situ* reconstruction techniques have been developed using new silver implants, rifampicin and arterial or venous homografts.

Results on the replacement of an infected graft with arterial or venous autografts in 24 patients have been first reported by Ehrenfeld et al. [11] in 1979. More recently, Clagett et al. [12] described the i*n situ* reconstruction with deep and superficial veins in 20 patients, the majority of them suffered from aortic prosthetic infection but there was aorta-enteric erosion and aorta-enteric fistula in one case each. The authors found a significantly higher failure rate after saphenous vein reconstruction compared to deep vein reconstruction. Nevelsteel et al. [13] reported on 15 patients with aortic prosthetic infection who underwent deep vein autograft repair. Thirteen patients had primary graft infection and only 2 patients had secondary aorta-enteric erosion. Thy concluded that the technique of deep venous reconstruction provides good potential for salvage of life and limbs in the management of prosthetic infection.

The technique of *in situ* reconstruction with autologous venous graft was used for the treatment of aortic prosthetic infection without fistula in the majority of patients reported by Clagett [12] and Nevelsteel [13]. In our series, the same technique was applied to a cohort of patients suffering from SAEF that is a life-threatening situation requiring urgent operation, and consequently there is a higher risk of failure than in the case of primary graft infection. Despite the limited number of patients, our results show that the *in situ* reconstruction technique with deep venous graft for treatment of SAEF is effective. To our knowledge, this is one of the largest single-center series of SAEF treated with venous homograft and observed for a very long follow-up. The in-hospital mortality (14%) was acceptable, only mild postoperative complications occurred, and no re-infections, occlusions or failures of the homograft have been documented at long-term follow-up. We preferred the use of the superficial femoral vein to create the graft because the large saphenous vein is often affected by significant intimal hyperplasia that can cause steno-occlusive complications [14].

Only recently, the use of endovascular techniques showed excellent short-term results for SAEF treatment, representing an alternative to open surgery or a “bridge” towards open surgery, after patient stabilization in cases of bleeding [9,15]. Unfortunately, long-term results are weighted by significant percentages of prosthetic re-infections [16,17]. Kakkos et al. [18] performed a review on management strategies for SAEF in 2016. They found that endovascular repair offers a better early survival than open surgery, but it is associated to a significantly higher rate of recurrent sepsis in the long-term follow-up. Therefore, a staged approach with early conversion to *in situ* repair, preferably with a venous graft, may achieve the best result. More recently, Heinola et al. [19] reviewed a series of 55 patients with aortic prosthesis infection treated by graft removal and *in situ* femoral veins replacement. Eighteen patients had SAEF, and the Authors suggest treating this condition with an aortic endograft as bridging technique only. They concluded that *in situ* reconstruction with femoral veins presents acceptable morbidity and mortality, and remains the treatment of choice for aortic graft infections at their hospital. A very recent meta-analysis on 402 patients (92 with SAEF) treated for aortic stent-graft infection after endovascular repair concluded that surgical treatment is a better option compared with conservative management [20]. Therefore, the open approach must be still kept in mind, in relation with the clinical conditions and life expectancy of the patient. Well-designed comparative studies may better clarify the optimal reconstructive technique for SAEF, however the very low incidence of this condition makes extremely difficult to organize a randomized trial.

In conclusion, the optimal management of SAEF is still unclear. Our results suggest that superficial femoral vein reconstruction of the abdominal aorta for SAEF is effective with an acceptable in-hospital mortality and low rate of major complications. We stress the importance of the deep femoral veins to create the graft because the large saphenous vein is often affected by significant intimal hyperplasia that can cause steno-occlusive complications.

## Funding

This research did not receive any specific grant from funding agencies in the public, commercial, or not-for-profit sectors.

## Provenance and peer review

Not commissioned externally peer reviewed.

## Ethical approval

An institutional board approved the study and informed consent was obtained from patients prior to each procedure.

## Sources of funding

This study was supported by institutional funding (University of Sassari).

## Author contribution

Feo CF wrote the paper.

Feo CF, and Ginesu GC designed the study.

Pinna A and Galotti F performed the research and acquired the data.

Paliogiannis P, Fancellu A and Porcu A analyzed the data and contributed to the revision of the manuscript.

## Trial registry number

Researchregistry5210.

## Guarantor

Claudio F Feo.

Panagiotis Paliogiannis.

## Declaration of competing interest

The authors declare that they have no conflict of interest.
